# Recovery Profiles and Well‐Being Outcomes in Patients Undergoing Various Anaesthesia Techniques: A Systematic Review

**DOI:** 10.1002/hsr2.70937

**Published:** 2025-06-20

**Authors:** Abdullah K. Bubshait

**Affiliations:** ^1^ Department of Anesthesia, College of Medicine Imam Abdulrahman Bin Faisal University Dammam Saudi Arabia

**Keywords:** anesthesia, anesthetics, hemodynamics, propofol, xenon

## Abstract

**Background and Aim:**

An appropriate anaesthesia technique is considered for the patient's health status to ensure a painless recovery. This study explored the important role of anesthesia selection in improving recovery outcomes and patient well‐being across diverse surgical settings.

**Methods:**

This study employed a systematic literature review methodology. Different databases, including Scopus, Google Scholar, and PubMed, were used to search for potential studies. A total of 20 studies were selected for qualitative analysis.

**Results:**

We found that general anesthesia is widely favored. However, certain procedures stressed the efficacy of spinal and regional approaches in managing pain and promoting faster recovery. Intravenous anesthetics such as propofol and newer agents like remimazolam and ciprofol are associated with improved recovery rates in high‐risk patients, providing hemodynamic stability and reducing the risk of postoperative nausea and vomiting. Furthermore, Opioid‐sparing and xenon‐based anesthetics also contributed to better recovery profiles.

**Conclusion:**

Anesthesia choice plays a critical role in patient recovery, with tailored techniques enhancing outcomes and reducing adverse effects.

## Summary

- For surgical procedures, a suitable anesthesia technique is used for quick recovery.
- Intravenous anesthetics, along with newer agents, lead to better recovery outcomes in high‐risk patients by minimizing side effects.
- The use of innovative techniques by incorporating new anesthetic agents can optimize patient well‐being outcomes.

## 1 Introduction

A successful surgery is executed when the patients are given importance for their well‐being and satisfaction in pre‐ and post‐surgical periods. Recent guidelines from the European Society of Anaesthesiology and Intensive Care (ESAIC) emphasize the critical importance of comprehensive preoperative assessment in selecting the most appropriate anesthesia technique, considering both clinical factors and patient preferences [1]. An appropriate anaesthesia technique, considering the patient's health status, ensures a painless recovery with quality.

The concept of Enhanced Recovery after Surgery (ERAS) was proposed by Henry Kehlet, advocating a multimodal strategy for perioperative care focused on accelerating recovery through a better understanding of the body's pathophysiological responses [2]. ERAS protocol led to the development of standardized protocols for the preoperative, intraoperative, and postoperative phases. Preoperative patient education as part of the ERAS protocol facilitates reducing anxiety and supporting faster restoration of the body's function after surgery [3]. Intraoperative fluid management targeting zero balance reduces morbidity, accelerates recovery, and shortens hospital stays while the risk of complications is reduced with early postoperative enteral feeding [4, 5]. The benefits of the ERAS protocol, including reduced costs, fewer complications, faster rehabilitation, and improved patient acceptance, have been extensively demonstrated across several surgical fields [6, 7]. Recovery is significantly accelerated by effective pain management since postoperative pain often leads to prolonged hospitalization, increased readmissions, and greater healthcare expenditures. Several multimodal analgesic strategies have been proposed to manage postoperative pain and reduce opioid use [8]. Among current approaches, the Transversus Abdominis Plane (TAP) block, introduced in 2001, is a regional anesthesia technique that delivers local anesthetic into the fascial plane between the internal oblique and transversus abdominis muscles. It primarily targets the lower thoracic spinal nerves (T7–T12) and the iliohypogastric and ilioinguinal nerves (L1) [9]. Rouholamin et al. [10] investigated the efficacy of TAP block and demonstrated that TAP block with ropivacaine 0.5% significantly minimizes the postoperative pain of laparoscopic surgery.

Nowadays, anaesthesiology has gained traction as a separate branch for assuring effective intraoperative management and the patient's postoperative well‐being. For pediatric cleft palate surgery, stable intraoperative hemodynamics, and analgesia are required for speedy recovery of children. These goals are attained when new techniques are formed and practiced by the anesthesiologists such as inhalational agents, dexmedetomidine, nerve blocks, total intravenous anesthesia (TIVA), local site infiltration, nonsteroidal anti‐inflammatory drugs, and opioids [11, 12]. Inadequate studies discussed the effects of long‐acting opioids on perioperative analgesia and hemodynamics in cleft palate surgery [13]. At tertiary care hospitals, the most commonly used drug for perioperative analgesia is buprenorphine in cleft palate surgery [14]. Besides, newer technologies and methods are in use in surgical techniques, pain management, and sedation for the perioperative care of patients [15]. The score of quality of recovery (QoR) is typically used for assessing the efficacy of these treatments as a gauge of patients' overall health following aesthetic‐assisted surgical operations [16]. QoR‐15, the most recent version, has high response and completion rates, a greater time efficiency rating, and both [17]. It is employed to verify to patients the efficacy of surgical procedures [18]. Propofol‐based total intravenous anesthesia (TIVA) has a higher QoR score for intravenous anesthesia than balanced inhalational anesthesia among patients who had surgical treatments over the last 10 years. The efficiency of propofol in reducing perioperative stress, physiologic deterioration, and inflammatory response may be the fundamental cause of this reduction in the QoR score [19]. Despite its effectiveness leading to high scores, injection pain, risk of fatal metabolic derangement (which is a rarely occurring disorder), and cardiorespiratory depression are some of the drawbacks of propofol‐based general anesthesia [20, 21].

The literature has mentioned many anaesthesia drugs and compared techniques to reduce the pain for a healthy recovery. The current study aims to gather evidence to provide publication bias and the emergence of anesthesia techniques over time to provide a comparison between the old techniques and new techniques for development. This will help the audience to understand the recent innovations in technology to provide a successful recovery for patients undergoing any form of surgery.

## 2 Methods

**Figure 1:**
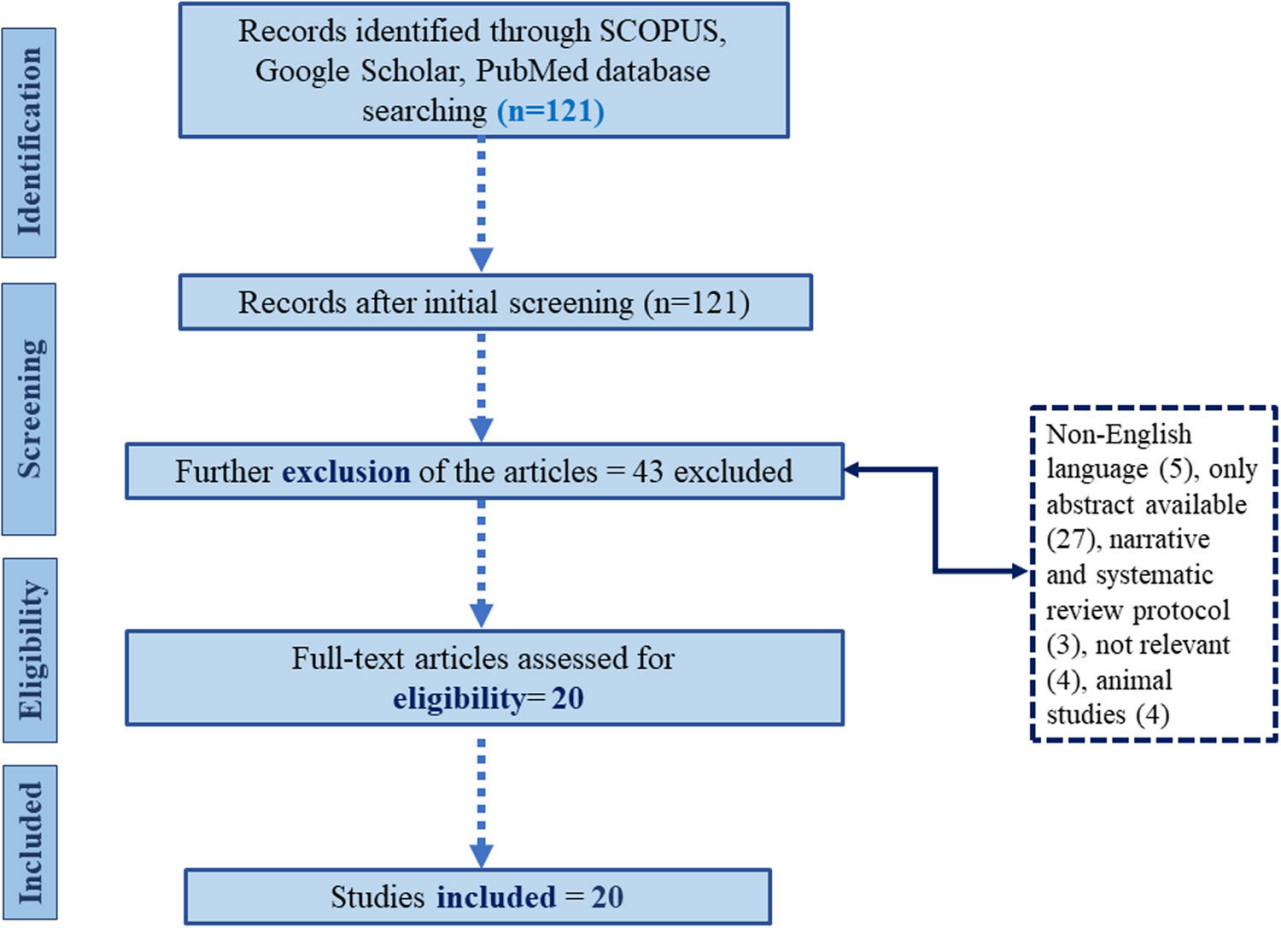
PRISMA framework.

This study conducted a systematic literature review in adherence with the Preferred Reporting Items for Systematic Reviews and Meta‐analysis (PRISMA) framework. The included studies were published from 1994 to 2024. PRISMA flowchart is presented in Figure 1 to illustrate study selection and exclusion.

### 2.1 Search Strategy

The systematic search of literature was carried out on three different databases such as Google Scholar, PubMed, and Scopus. The keywords used for the search strategy are as follows; [“Anaesthesia” OR “general anesthesia” OR “spinal anesthesia” OR “local anesthesia” OR total intravenous anesthesia” OR “TIVA”] AND [“recovery profile” OR “quality of recovery” OR “QoR” OR “well‐being” OR “postoperative recovery”].

### 2.2 Inclusion and Exclusion Criteria

The criteria for inclusion required studies to be original research, focusing on randomized controlled trials, and observational and retrospective studies. The literature that discussed the effectiveness and the adoption of anesthesia was considered eligible. The studies that didn't adopt anesthesia as an agent in the processes of surgery were excluded. Further, studies were excluded based on the following reasons: full‐text not available, other languages than English, narrative and systematic reviews, and involved animal studies.

### 2.3 Data Extraction

Two authors performed independent data extraction using a standardized form, including the author's first name, study population and location, study design, type of anesthesia and medication volume, ASA score, type of surgery, and recovery outcomes. Any disagreements were resolved by the corresponding author. Of the 381 studies initially reviewed, 121 were relevant to the study topic. After removing duplicates, 83 studies remained. Following further filtering based on abstract, title, keywords, and eligibility criteria, 20 studies were selected.

### 2.4 Quality Assessment Tools

To assess the quality of selected studies, we used two quality assessment tools including the Cochrane risk of a bias assessment tool for randomized control trials and the Newcastle Ottawa scale for non‐randomized studies. The Cochrane risk of bias assessment tool is employed to assess the risk of bias (low or high) by focusing on key aspects such as randomization procedure, personnel and participants blinding, allocation concealment, outcome assessment blinding, missing data bias, and selective reporting bias. In addition, if the study lacks enough information, we classified the risk of bias as “unclear”. The Newcastle Ottawa scale assessed the quality of non‐randomized studies based on three aspects: selection, comparability, and exposure. This scale gives a maximum score of 4 for selection, 3 for exposure, and 2 for comparability, yielding a total possible score of 9. Studies scoring 5 or more are considered moderate to high quality, while those scoring less than 5 are rated as low quality.

## 3 Results

### 3.1 Study Selection

The publication's search for a systematic literature review identified a total of 121 studies through databases such as Google Scholar and Scopus. After initial screening and removing duplicate records (*n* = 58), the abstracts and titles of the studies (*n* = 63) were reviewed and a further 43 records were eliminated based on the following reasons; full‐text articles were not available (*n* = 27), non‐English language (*n* = 5), studies not incorporating anesthesia as the main outcome (*n* = 4), narrative and systematic reviews (*n* = 3), studies involved animals (*n* = 4). Therefore, a total of 20 studies published from 1994 to 2024 were incorporated into the qualitative analysis.

### 3.2 Risk of Bias

Overall, an analysis of randomized studies shows a low risk of bias, although it was unclear in the domain of allocation concealment and blinding of outcome assessment (Table 1). Despite this, the primary outcome had a low risk of bias, as it was not significantly influenced by blinding of outcome assessment and allocation concealment. Consequently, the risk assessment of non‐randomized studies is also presented which demonstrates that the majority of the studies exceeded the threshold score, indicating high quality (Table 2).

**Table 1:** Cochrane risk of bias assessment tool for randomized control trials.

Studies	Randomization process	Personal and participants blinding	Allocation concealment	Blinding of outcome assessment	Incomplete data outcome	Selective reporting	Overall bias
Bakhshi [22]	L	L	L	U	L	L	Low
Gao et al. [23]	L	U	U	U	L	L	Unclear
Hofer et al. [24]	L	L	L	L	L	L	Low
Cremer et al. [25]	L	L	U	L	L	L	Low
Moro et al. [26]	L	L	L	L	L	L	Low
Rortgen et al. [27]	L	H	L	L	L	L	Moderate
Singh et al. [28]	L	L	L	L	L	L	Low
Fredman et al. [29]	L	H	L	L	L	L	Moderate
Martikainen et al. [30]	L	H	U	L	L	L	Moderate
Nam et al. [31]	L	L	L	L	L	L	Low
Cheng et al. [32]	L	L	L	L	L	L	Low
Li et al. [33]	L	L	L	L	L	L	Low
He et al. [34]	L	L	L	L	L	L	Low
Gupta et al. [35]	L	L	L	L	L	L	Low

Abbreviations: H = high, L = low, U = unclear.

**Table 2:** The Newcastle Ottawa scale quality assessment tool for non‐randomized studies.

Studies	Selection (max 4)	Comparability (max 2)	Outcome (max 3)	Total (max 9)	Remarks
Engelman [36]	4	1	3	8	High
Ohkushi et al. [37]	4	2	3	9	High
Nilsson et al. [38]	4	1	3	8	High
Yu et al. [39]	2	1	3	6	Moderate
Liu et al. [40]	3	2	3	8	High
Duran et al. [41]	3	1	3	7	Moderate

### 3.3 Study Characteristics

The primary attributes of 20 selected studies are outlined (Table 3). Six studies were performed in China [23, 32, 33, 34, 40, 41], two studies in Germany [25, 27], and one study in the United Kingdom [22], United States [36], Japan [37], Sweden [38], Switzerland [24], Brazil [26], India [28], Israel [29], Finland [30], South Korea [31], Turkey [41]. Consequently, these studies recruited a total of 3069 subjects, and the size of the sample of an individual record varies from 20 to 562 patients. The participants' age ranged from 18 to 85 years, and they were predominantly male. Most of the studies enrolled patients with American Society of Anaesthesiologists (ASA) physical status I‐II while three studies [25, 27, 29] enrolled participants with ASA status I‐III and one study involved patients with ASA status I. A broad range of surgical interventions were conducted in the studies, reflecting multiple approaches and specialties. The majority of the studies used general anesthesia [22, 23, 24, 25, 26, 27, 28, 32, 33, 34, 39, 41] while two studies involved local anesthesia [37, 38] and three studies incorporated spinal anesthesia [23, 29, 30].

**Table 3:** Characteristics of selected studies.

Author	Title	Country	Number of participants (gender)	Type of study	Age	ASA status	Type of surgery	Type of anesthesia	Anesthesia medication (total volume)	Recovery profile
Bakhshi [22]	An open randomized study of the effects of intravenous fluid replacement during the day case anesthesia	United Kingdom	66 (Female)	Open randomized study	17–30	I	Therapeutic termination of pregnancy	General anesthesia	Fentanyl (1 µg/kg), propofol (2.0–2.5 mg/kg). Anaesthesia was maintained with 5 mg incremental boluses of propofol.	This study found that propofol promotes a good quality of recovery and a low rate of nausea.
Engelman [36]	Mechanisms to reduce hospital stays	United States	562 patients (predominantly male)	6‐month retrospective analysis	65 years (average)	—	Fast track surgery	Not specified	Sufentanil (5 µg/kg), Fentanyl (20– 25 µg/kg), and Midazolam (0.15 mg/kg).	This study revealed that in fast‐track surgery, anesthesia plays a contributing role in recovery and early discharge from the ICU.
Gao et al. [23]	Effect of remimazolam versus propofol for the induction of general anesthesia on cerebral blood flow and oxygen saturation in elderly patients undergoing carotid endarterectomy	China	43 patients (predominantly male); Remimazolam group (*N* = 21), propofol group (*n* = 22)	Randomized clinical trial	60–75 years	≥ V	Carotid endarterectomy	General anesthesia	Remimazolam (0.3 mg/kg), Propofol (1.5–2 mg/kg). An additional dose was administered with 2.5 mg remimazolam or 50 mg propofol if the BIS > 60 after 3 min. “Sufentanil (3 µg/kg) and *cis*‐atracurium (2 mg/kg) were administered after achieving a BIS ≤ 60.	This study demonstrated that the administration of remimazolam could be effective and safe during general anesthesia induction and could improve hemodynamic parameters as compared to propofol.
Ohkushi et al. [37]	Recovery profile and patient satisfaction after ambulatory anesthesia for dental treatment—A crossover comparison between propofol and sevoflurane	Japan	20 patients (both)	Comparison study	35.1 (average)	I or II	Dental caries	Ambulatory anesthesia	Induction of anesthesia with 1% Diprivan injection in the propofol group and 3% of sevoflurane in the sevoflurane group. The propofol dose was increased by 0.2 µg/mL, and sevoflurane was increased by 0.5% 0.2 mg/kg when body movement was observed. An additional dose of 0.2 mg/kg rocuronium bromide was given if movement persisted.	Patients reported increased satisfaction and a stronger preference for subsequent dental treatments when propofol anesthesia was administered. This implies that propofol may be a better option for ambulatory anesthesia in dental care.
Nilsson et al. [38]	Postoperative recovery after general and regional anesthesia in patients undergoing day surgery: A mixed methods study	Sweden	401 patients (both)	Mixed methods study	Above 17 years	—	General, hand, orthopedic surgery	General and regional anesthesia	—	This study reported that patients who underwent general anesthesia showed poorer recovery than regional anesthesia. The major observed problem was the pain in the surgical wounds, following tiredness and fatigue.
Hofer et al. [24]	Patient well‐being after general anesthesia: A prospective, randomized, controlled multi‐center trial comparing intravenous and inhalation anesthesia	Switzerland	305 patients, (predominantly female); TIVA group (*n* = 155), INHAL group (*n* = 146)	Prospective, randomized, controlled multi‐center trial	20–79 years	I or II	Minor elective gynecologic or orthopedic interventions	General anesthesia	Fentanyl (3 µg/kg), lidocaine 1% (10 mg) and propofol (1.4 mg/kg). Rocuronium (0.6 mg/kg) was administered for intubation. Anesthesia maintained with propofol infusion (2–8 mg kg^−1^ h^−^^1^ or inhaled sevoflurane	Total inhaled volatile anesthesia enhances postoperative recovery outcomes and lowers the risk of postoperative nausea and vomiting.
Cremer et al. [25]	Early cognitive function, recovery, and well‐being after sevoflurane and xenon anesthesia in the elderly: A double‐blinded randomized controlled trial	Germany	40 patients (both); Sevoflurane group (*n* = 20), Xenon group (*n* = 19)	A double‐blinded randomized controlled trial	65–75 years	I–III	Elective surgery (urology, gynecology, neurosurgery, trauma, ENT, orthopedics, and abdominal surgery)	General anesthesia	Propofol (2 mg/kg), Remifentanil (0.5 μg/kg over 60 s), and Rocuronium (0.6 mg/kg). Anaesthesia was maintained either with Xenon (60% xenon in 30% oxygen) or sevoflurane (1.1–1.4 vol% in 30% oxygen).	This study reported no significant difference in the occurrence of postoperative cognitive dysfunction across the two groups. However, the fastest recovery was observed in the xenon group.
Moro et al. [26]	Quality of recovery from anesthesia of patients undergoing balanced or total intravenous general anesthesia. Prospective randomized clinical trial	Brazil	130 patients (both); intravenous anesthesia group (*n* = 54), inhalation anesthesia group (*n* = 56)	Prospective randomized clinical trial	18–65 years	I–II	Otorhinolaryngological surgery	General anesthesia	Midazolam 0.06 mg/kg, 1% lidocaine (30 mg). Both groups: remifentanil, induction dose (0.5 μg/kg/min for 3 min, followed by a maintenance dose of 0.3 μg/kg/min. For Group V: Propofol, initial bolus (2.0 mg/kg) followed by infusion at 4–6 mg/kg/h Group I: Propofol bolus (2.0 mg/kg), maintenance with 2% sevoflurane in O_2_/air flow (fraction of inspired oxygen, 60%) 2 L/min.	The assessment of the QoR‐40 questionnaire showed no difference in the quality of recovery from anesthesia, as perceived by patients, between those treated with intravenous or inhalation general anesthesia for otorhinolaryngological surgery.
Rortgen et al. [27]	Comparison of early cognitive function and recovery after desflurane or sevoflurane anesthesia in the elderly: A double‐blinded randomized controlled trial	Germany	80 patients, (not specified); e Desflurane group (*n* = 40), Sevoflurane group (*n* = 40)	A double‐blinded randomized controlled trial	65–85 years	I–III	Elective surgery in traumatology, ear, nose, and throat surgery, gynecology, urology, or neurosurgery	General anesthesia	Propofol (2 mg/kg), Remifentanil (0.5 µg/kg) over 60 s, and Rocuronium (0.6 mg/kg). Anesthesia was maintained using age‐adjusted MAC levels of desflurane (4.2%–4.5%) or sevoflurane (1.1%–1.4%) in 30% oxygen	This study reported no difference in the total occurrence of postoperative cognitive dysfunction between the two groups. In contrast, desflurane showed better outcomes in the Trail Making Test, well‐being scale test, patient satisfaction, and emergence times.
Singh et al. [28]	Comparison of recovery profile for propofol and sevoflurane anesthesia in cases of open cholecystectomy	India	60 patients, (both); Sevofl urine anesthesia group (*n* = 30), propofol infusion group (*n* = 30)	Prospective, randomized, and blinded, clinically controlled study	18–65 years	I and II	Elective cholecystectomy	General anesthesia	In both groups, induction was done with Propofol (2 mg/kg) and after induction, patients were maintained on N_2_O/O_2_ (60%/40%) and Propofol infusion (75–125 μg/kg/min) (group 1), and Sevoflurane anesthesia (1%–2%) (group 2)	This study found that Propofol as well as sevoflurane both were good for the maintenance of anesthesia in open cholecystectomy surgery.
Fredman et al. [29]	The induction, maintenance, and recovery characteristics of spinal vs. general anesthesia in elderly patients	Israel	100 elderly patients, (both); P–P group (*n* = 25), P–I group (*n* = 25), P–D group (*n* = 25), S group (*n* = 25)	Randomized, prospective, open‐label study	62–82 years	I/II/III	Transurethral resection of bladder tumor surgery, transurethral resection of the prostate surgery	General and spinal anesthesia	P–P GA group received (propofol‐induced at 1.0–2.0 mg/kg IV and maintained at 75– 150 mg/kg/min. P–D GA group (propofol‐induced at 1.0–2.0 mg/kg and desflurane (end‐tidal 1%–4%) for maintenance. P–I GA group: Propofol induced at 1.0–2.0 mg/kg and isoflurane (end‐tidal 0.7%–1.2%) for maintenance, in the S group 1.5 mL 4% lidocaine (60 mg) was in an equal volume of 10% dextrose water. In Groups P–P, P–D, and P–I, patients received fentanyl 1–2 mg/kg IV and breathed 100% oxygen for 2–3 min before induction of anesthesia.	It is observed that Propofol and desflurane‐based general anesthesia offers shorter induction and recovery times, without negatively impacting patient comfort. This makes it a preferable choice over spinal anesthesia for elderly patients undergoing short transurethral surgeries.
Martikainen et al. [30]	One‐week recovery profiles after spinal, propofol, isoflurane, and desflurane anesthesia in ambulatory knee arthroscopy	Finland	173 pateints (both); Propofol group (*n* = 32), Isoflurane group (*n* = 38), Desflurane group (*n* = 48), spinal group (*n* = 55)	Randomized control trial	18–65 years	I or II	Elective knee arthroscopy	General and spinal anesthesia	SA was given with lidocaine 50 mg/mL). The Propofol group was anesthetized with propofol (bolus 2 mg/kg, followed by a continuous infusion of 1 of 2 mg/kg/h for 15 min, then 9 mg/kg/h for 15 min, and 6 mg/kg/h as needed until surgery end), Isoflurane (titrated up to 1 MAC before skin incision), The desflurane group received desflurane after the same propofol induction dose.	General anesthesia could be a suitable option in terms of patient comfort.
Nam et al. [31]	Effects of opioid‐sparing general anesthesia on postoperative nausea and vomiting in laparoscopic gynecological surgery	South Korea	120 patients, (not specified); OSA group (*n* = 60), OUA group (*n* = 60)	Prospective, randomized controlled study	43.7 years (average)	I or II	Elective laparoscopic gynecological surgery	General anesthesia	Anesthesia induction with thiopental so—medium (5 mg/kg), sevoflurane (6 vol%), and rocuronium (6 mg/kg), for both groups. While for group OUA, targeting remifentanil (3 ng/mL). Anesthesia was maintained with sevoflurane under BIS monitoring, targeting a range between 40 and 60.	This study found that OSA significantly reduced postoperative nausea, and pain, with no increase in hemodynamic instability in patients undergoing laparoscopic gynecological surgery.
Cheng et al. [32]	Efficacy and safety of remimazolam tosilate in anesthesia for short otolaryngology surgery	China	85 patients, Remimazolam (RM) group (*n* = 42), Midazolam (MD) group (*n* = 43)	Unicentric, double‐blind, randomized controlled study	18–60 years	I–II	Otolaryngology surgery	General anesthesia	Sedation induced with Remimazolam tosilate (0.3 mg/kg) or Midazolam (0.075 mg/kg) to RM and MD group, respectively. anesthesia was maintained with propofol (4–12 mg/kg/h) and remi fentanil (3–120 µg/kg/h)combined with sevoflurane (1%–2%) inhalation.	This study observed that for short otolaryngology surgeries, remimazolam tosilate shows efficacy, promoting a rapid onset, faster post‐surgery recovery, lower rate of perioperative adverse events, and higher satisfaction as compared to midazolam.
Li et al. [33]	The efficacy and safety of ciprofol vs. propofol in patients undergoing painless hysteroscopy: A randomized, double‐blind, controlled trial	China	217 participants; Ciprofol group (*N* = 109), Propofol group (*n* = 108).	A double‐blind randomized controlled trial	Above 18 years old	I–II	Hysteroscopy	General anesthesia	Alfentanil (5 µg/kg) was used for 30 s for general anesthesia induction for both groups. Experimental group: Injected with ciprofol (0.4 mg/kg). The control group: was injected with propofol (2 mg/kg).	It was found that ciprofol is as efficient as propofol for general anesthesia during painless hysteroscopy.
He et al. [34]	Efficacy and safety of Ciprofol vs. Propofol as an anesthetic for patients undergoing painless colonoscopy	China	222 patients; Propofol group (*n* = 112), Ciprofol group (*n* = 110).	Randomized, double‐blind controlled study	18–65 years	I–II	Colonoscopy	General anesthesia	Sufentanil (0.05 μg/kg) administered before IV infusion of either Ciprofol (0.4 mg/kg) or Propofol (2 mg/kg) over 1 min for induction.	This study revealed that ciprofol induction is associated with improved hemodynamic stability and less injection pain compared to propofol for anesthesia in patients undergoing painless colonoscopy.
Yu et al. [39]	Analysis of postoperative nausea and vomiting in patients with lung cancer undergoing thoracoscopic surgery under general anesthesia and its influencing factors: An observational study	China	200 patients	Observational study	Above 18 years	I–II	Thoracoscopic lung cancer surgery	General anesthesia	—	This study observed postoperative nausea and vomiting in patients undergoing thoracoscopic lung cancer surgery with general anesthesia.
Liu et al. [40]	Comparison of spinal anesthesia and local anesthesia in percutaneous interlaminar endoscopic lumbar discectomy (IELD) for L5/S1 disc herniation: A retrospective cohort study	China	115 patients (both); local anesthesia group (*n* = 56), spinal anesthesia group (*n* = 59)	Retrospective cohort study	18–70 years	I–II	Lumbar disc herniation	Local and spinal anesthesia	The LA group administered 1% lidocaine. In the SA group o1% lidocaine and 2.0 mL of (0.5%) Ropivacaine to ensure aseptic technique.	Spinal anesthesia exhibited effectiveness in IELD surgery as compared to local anesthesia but the possible risk related to this type of anesthesia must be evaluated. As an alternative anesthesia for IELD surgery holds great promise, exhibiting superior efficacy compared to LA. However, it is crucial to meticulously evaluate the indications due to potential risks associated with this form of anesthesia.
Duran et al. [41]	The effect of two different modes of anesthesia maintenance on postoperative delirium in elderly patients with low preoperative mini‐cog score	Turkey	84 patients (both); Sevoflurane group (*n* = 41) and TIVA group (*n* = 43)	Prospective observational study	Over 60 years	Patients were preoperatively assessed for ASA risk by an anesthetist	Laparoscopic cholecystectomy	General anesthesia	Propofol (2 mg/kg), fentanyl (2 μg/kg), and rocuronium (0.6 mg/kg) were used intravenously for anesthesia induction. In the TIVA protocol, induction was achieved with propofol (0.1–0.2 mg/kg/min) and remifentanil (0.1 0.2 mg/kg/min) while sevoflurane protocol with 25 sevoflurane.	It is observed that postoperative delirium is more associated with the use of sevoflurane compared with that of TIVA.
Gupta et al. [35]	Assessment of recovery following day‐case arthroscopy	Not mentioned	50 healthy patient (both); Isofluranegroup (*n* = 26), Propofol group (*n* = 24)	Double‐blind randomized study	15–45 years	I or II	Day‐case arthroscopy	General anesthesia	Propofol group: propofol (10 mg. kg^−1^ h^−1^ for 5 min) then until the end of the procedure (6 mg. kg^−1^. h) Isoflurane group: Isoflurane (0.5%–2%). In both groups, alfentanil 0.25 mg was given before anesthesia induction, before incision, and every 15 min thereafter until surgery ended.	This study revealed that isoflurane‐based anesthesia showed better recovery of psychomotor parameters when administered for day‐case arthroscopy. However, all factors (discharge and awakening time) were the same for both approaches.

### 3.4 Anaesthesia Types and Recovery Outcomes

#### 3.4.1 General Anaesthesia

Previous studies have explored the influence of different general anesthesia protocols on patients' recovery outcomes. Bakhshi [22] argued that propofol, coupled with fentanyl, stimulates recovery quality and lowers the rate of postoperative nausea. Engelman [36] found that general anesthesia in fast‐track surgery contributed significantly to patients' quick recovery and earlier ICU discharge. Gao et al. [23] demonstrated that the use of remimazolam during general anesthesia improves hemodynamic parameters more than the use of propofol. Nam et al. [31] evaluated opioid‐sparing general anesthesia with remifentanil, demonstrating that its use considerably minimized postoperative pain and nausea without compromising hemodynamic stability. Singh et al. [28] found no significant difference in recovery quality of propofol and sevoflurane, while Rortgen et al. [27] demonstrated higher patient satisfaction and better cognitive outcomes with desflurane, as compared to sevoflurane.

#### 3.4.2 Spinal and Local Anaesthesia

Several studies compared spinal anesthesia with other types of anesthesia. Fredman et al. [29] advocated that propofol and desflurane‐based general anesthesia were preferred due to faster recovery times compared to spinal anesthesia. Martikainen et al. [30] demonstrated that spinal anesthesia promotes a good recovery profile while general anesthesia offers better patient satisfaction. Liu et al. [40] found that local anesthesia is not as effective as spinal anesthesia in lumbar disc surgery.

## 4 Discussion

This systematic literature review examined the well‐being outcomes and recovery profile connected with different anesthesia techniques, emphasizing the key influence of anesthesia choice on recovery. This study discussed the evidence from the selected studies, elucidating the variation in recovery and patient well‐being across different anesthesia techniques. It is observed that general anesthesia is the most preferable anesthesia technique as the majority of the studies used this type of anesthesia [22, 23, 24, 25, 26, 27, 28, 32, 33, 39, 41] while local anesthesia was used in one of the studies [37]. However, included studies had heterogeneity in terms of differences in participants (age, gender, and geographic location), interventions (variation in dosages and types of anesthesia), study design (randomized controlled trials, retrospective study, cohort studies, or case‐control studies), and sample sizes.

In comparison to spinal anesthesia, Fredman et al. [29] and Martikainen et al. [30] advocated that general anesthesia is a better option, particularly in ambulatory surgery and transurethral surgical procedures, respectively. Liu et al. [40] found that spinal anesthesia offered more effective analgesia than local anesthesia during lumbar disc herniation surgeries, and careful evaluation is required for its associated possible risks. Consequently, Nilsson et al. [38] found regional anesthesia is more effective as compared to general anesthesia, offering fatigue reduction and the fastest recovery. This implies a trade‐off between speedy recovery and constant pain control, where the choice of anesthesia should support the patient's recovery priorities and surgical requirements.

Intravenous anesthesia, specifically using agents like propofol, was linked with better recovery profiles in several studies. Bakhshi [22] advocated that propofol‐based general anesthesia promotes a high quality of recovery with minimal postoperative nausea in day‐case surgeries. Similarly, Lakra et al. [14] exhibit that in cleft palate surgeries single‐dose buprenorphine and propofol as the best anesthesia agents for a successful recovery and alleviating postoperative pain. Engelman [36] supported these results in the context of fast‐track surgery, where intravenous anesthesia agents like sufentanil and midazolam, enabled early discharge from the ICU and decreased the length of hospital stays. The rapid action of intravenous agents makes them highly appropriate for surgeries necessitating quick patient turnover and minimal postprocedure sedation. Further, Joe et al. [42] reported total intravenous anesthesia (TIVA) as a better option for patients recovering after curative pancreatectomy. Besides, traditional agents, emerging intravenous anesthesia agents, including remimazolam and ciprofol, have exhibited potential. Gao et al. [23] reported that remimazolam enhanced hemodynamic stability and offered effective induction of general anesthesia to elderly patients who underwent carotid endarterectomy. However, Choi et al. [21] compared the effectiveness of remimazolam‐based total intravenous anesthesia and propofol and found propofol was less effective in recovery after surgery as compared to the other. Correspondingly, studies by Li et al. [33] and He et al. [34] emphasized the benefit of ciprofol over propofol in pain reduction at the injection site and improved hemodynamic stability during procedures of painless hysteroscopy and colonoscopy. These findings suggest that the administration of intravenous anesthesia agents could further refine recovery outcomes, specifically in high‐risk populations.

On the other hand, previous studies also frequently compared the efficiency of intravenous anesthesia agents with inhalation anesthetics agents, particularly sevoflurane and desflurane. Hofer et al. [24] found that total intravenous anesthesia (TIVA) with propofol infusion lowered the risk of postoperative nausea and vomiting (PONV) compared to inhalational anesthesia with sevoflurane. These results highlighted that intravenous techniques may offer a distinct advantage. Similarly, Ohkushi et al. [37] reported that patients expressed higher satisfaction and preference for propofol over sevoflurane in dental ambulatory procedures. However, Singh et al. [28] observed favorable recovery profiles with both propofol and sevoflurane during open cholecystectomy surgeries, but broader acceptance of propofol among patients underscores the need to take subjective experiences into account in the process of anesthesia selection.

Furthermore, the study of Rortgen et al. [27] highlighted specific benefits of inhalational agents, demonstrating that desflurane outperformed sevoflurane in cognitive recovery metrics and patient satisfaction in elderly patients undergoing elective surgeries. In contrast, Moro et al. [26] evaluated recovery quality through the QoR‐40 questionnaire and observed no significant differences between intravenous and inhalation anesthesia in patients undergoing otorhinolaryngological surgeries. This suggests that both techniques seem equally feasible for broader recovery metrics. While Fredman et al. [29] advocated that propofol and desflurane‐based general anesthesia promote shorter induction and recovery times. In addition, Cremer et al. [25] compared the use of xenon and sevoflurane in elderly patients and observed that xenon offered faster recovery times with a low risk of postoperative cognitive dysfunction (POCD). Similarly, Nam et al. [31] explored opioid‐sparing anesthesia (OSA) protocols, which significantly lowered postoperative nausea and vomiting, and pain scores, with hemodynamic stability in laparoscopic gynecological surgeries.

The results of our study support the principles of ERAS protocols, advocating for fat recovery strategies that enable early mobilization, minimize opioid dependence, and reduce postoperative complications. Agents including ciprofol and remimazolam have shown benefits not only in hemodynamic stability but also in improving patient comfort and minimizing recovery time, which are primary purposes in patient‐centered anesthesia.

This systematic review explored the important role of anesthesia selection in improving recovery outcomes and patient well‐being across diverse surgical settings. General anesthesia emerged as the most commonly used technique, however, specific procedures underscored the benefits of spinal and regional anesthesia for pain control and rapid recovery. Intravenous agents like propofol and emerging options such as remimazolam and ciprofol were associated with improved recovery profiles, particularly in high‐risk populations, demonstrating hemodynamic stability and minimized rate of postoperative nausea and vomiting. Innovative approaches such as opioid‐sparing protocols and the use of xenon further improved recovery by minimizing common side effects. The results underscore the need to align anesthesia selection with a patient‐centric approach, guided by surgical demands and recent innovations, to achieve optimal recovery and mitigate possible risks.

## 5 Clinical Implications

This study provides significant clinical implications for anesthesia selection on postoperative recovery and patient well‐being, underscoring the necessity for fast recovery strategies and patient‐centered anesthesia planning in surgical care. General anesthesia continues to be the most commonly employed technique, owing to its effectiveness across multiple surgical disciplines. Nonetheless, regional anesthesia has been demonstrated to be an effective alternative when the primary target is to minimize recovery time and effective pain relief, particularly in orthopedic procedures. Clinicians should focus on the potential benefits of accelerated recovery and minimized postoperative fatigue when choosing anesthesia methods. Total intravenous anesthesia (TIVA), particularly with agents like propofol, remimazolam, and ciprofol shows the potential to enhance anesthesia safety and effectiveness. Clinicians should consider the use of these agents specifically in high‐risk groups (the elderly or patients undergoing complex surgeries) to minimize hemodynamic fluctuations or cognitive impairment. In addition, the emergence of opioid‐sparing anesthesia and novel agents such as Xenon advocates an increasing emphasis on individualized, patient‐centered anesthesia care that balances reduced side effects with effective recovery. Clinicians should integrate opioid‐sparing anesthesia as these strategies support better pain management, earlier discharge, and higher patient satisfaction. Given the significant impact of anesthesia on postoperative outcomes, incorporating standardized, evidence‐based anesthesia protocols into ERAS programs surgical outcomes, patients' well‐being, and faster patient recovery and discharge.

## Author Contributions

**Abdullah K. Bubshait:** conceptualization, methodology, validation, visualization, funding acquisition, resources, supervision, data curation, formal analysis, writing – original draft, writing – review and editing.

## Conflicts of Interest

The author declares no conflicts of interest.

## Transparency Statement

The lead author, Abdullah K. Bubshait, affirms that this manuscript is an honest, accurate, and transparent account of the study being reported; that no important aspects of the study have been omitted; and that any discrepancies from the study as planned (and, if relevant, registered) have been explained.

## Data Availability

Data sharing is not applicable to this article as no data sets were generated or analyzed during the current study.

## References

[1] M. Lamperti , C. S. Romero , F. Guarracino , et al., “Preoperative Assessment of Adults Undergoing Elective Noncardiac Surgery: Updated Guidelines From the European Society of Anaesthesiology and Intensive Care,” European Journal of Anaesthesiology|EJA 42, no. 1 (January 2025): 1–35. 10.1097/EJA.0000000000002069 39492705

[2] S. Forte , F. A. Ferrari , H. S. Majd , F. Cisotto , and F. Ferrari , “Enhanced Recovery After Surgery (ERAS) In Gynecology: State of the Art and the Problem of Barriers,” Clinical and Experimental Obstetrics & Gynecology 50, no. 1 (2023): 14.

[3] P. H. E. Teeuwen , R. P. Bleichrodt , C. Strik , et al., “Enhanced Recovery After Surgery (ERAS) Versus Conventional Postoperative Care in Colorectal Surgery,” Journal of Gastrointestinal Surgery 14, no. 1 (2010): 88–95. 19779947 10.1007/s11605-009-1037-x PMC2793377

[4] V. Nisanevich , I. Felsenstein , G. Almogy , C. Weissman , S. Einav , and I. Matot , “Effect of Intraoperative Fluid Management on Outcome After Intraabdominal Surgery,” Anesthesiology 103, no. 1 (July 2005): 25–32. 15983453 10.1097/00000542-200507000-00008

[5] S. J. Lewis , H. K. Andersen , and S. Thomas , “Early Enteral Nutrition Within 24 h of Intestinal Surgery Versus Later Commencement of Feeding: A Systematic Review and Meta‐Analysis,” Journal of Gastrointestinal Surgery 13, no. 3 (March 2009): 569–575. 18629592 10.1007/s11605-008-0592-x

[6] F. Ferrari , S. Forte , N. Sbalzer , et al., “Validation of An Enhanced Recovery After Surgery Protocol in Gynecologic Surgery: An Italian Randomized Study,” American Journal of Obstetrics and Gynecology 223, no. 4 (October 2020): 543.e1–543.e14. 10.1016/j.ajog.2020.07.003 32652064

[7] S. P. Bisch , C. A. Jago , E. Kalogera , et al., “Outcomes of Enhanced Recovery After Surgery (ERAS) in Gynecologic Oncology—A Systematic Review and Meta‐Analysis,” Gynecologic Oncology 161, no. 1 (April 2021): 46–55. 33388155 10.1016/j.ygyno.2020.12.035

[8] Q. Cai , M. Gao , G. Chen , and L. Pan , “Transversus Abdominis Plane Block Versus Wound Infiltration With Conventional Local Anesthetics in Adult Patients Underwent Surgery: A Systematic Review and Meta‐Analysis of Randomized Controlled Trials,” BioMed Research International 2020, no. 1 (2020): 8914953. 32280705 10.1155/2020/8914953 PMC7125448

[9] F. A. Ferrari , B. Crestani , L. Torroni , et al., “Wound Infiltration With Local Anesthetics Versus Transversus Abdominis Plane Block for Postoperative Pain Management In Gynecological Surgery: A Systematic Review and Meta‐Analysis of Randomized Controlled Trials,” Journal of Minimally Invasive Gynecology 32, no. 3 (2024): 229–239.e3, https://pubmed.ncbi.nlm.nih.gov/39510498/. 39510498 10.1016/j.jmig.2024.10.030

[10] S. Rouholamin , A. Ghahiri , and B. Dehghan Khalili , “The Efficacy of Ropivacaine 0.5% in Transversus Abdominis Plane Block to Relieve the Postoperative Pain of Female Laparoscopic Surgery Grade II,” Advanced Biomedical Research 11, no. 1 (January 2022): 12. 35386544 10.4103/abr.abr_46_20 PMC8977607

[11] L. E. Moggi , T. Ventorutti , and R. D. Bennun , “Cleft Palate Repair: A New Maxillary Nerve Block Approach,” Journal of Craniofacial Surgery 31, no. 6 (September 2020): 1547–1550. 32604288 10.1097/SCS.0000000000006633

[12] T. Flowers and R. Winters , “Postoperative Pain Management in Pediatric Cleft Lip and Palate Repair,” Current Opinion in Otolaryngology & Head and Neck Surgery 29, no. 4 (August 2021): 294–298. 34183559 10.1097/MOO.0000000000000719

[13] S. Fenlon and N. Somerville , “Comparison of Codeine Phosphate and Morphine Sulphate in Infants Undergoing Cleft Palate Repair,” Cleft Palate Craniofacial Journal 44, no. 5 (September 2007): 528–531. 10.1597/06-206.1 17760494

[14] P. R. Lakra , P. Thaware , and Bharati , “Assessment of Intraoperative Hemodynamics and Recovery Characteristics in Pediatric Patients Receiving Buprenorphine and Propofol Anesthesia for Cleft Palate Surgery: A Prospective Observational Study,” Anesthesia Essays & Researches 16, no. 2 (April 2022): 255–262. 36447914 10.4103/aer.aer_95_22 PMC9701334

[15] J. Kleif , J. Waage , K. B. Christensen , and I. Gögenur , “Systematic Review of the QoR‐15 Score, a Patient‐ Reported Outcome Measure Measuring Quality of Recovery After Surgery and Anaesthesia,” British Journal of Anaesthesia 120, no. 1 (January 2018): 28–36. 29397134 10.1016/j.bja.2017.11.013

[16] W. K. Lee , M. S. Kim , S. W. Kang , S. Kim , and J. R. Lee , “Type of Anaesthesia and Patient Quality of Recovery: A Randomized Trial Comparing Propofol–Remifentanil Total I.V. Anaesthesia With Desflurane Anaesthesia,” British Journal of Anaesthesia 114, no. 4 (April 2015): 663–668. 25500679 10.1093/bja/aeu405

[17] N. J. Pastis , L. B. Yarmus , F. Schippers , et al., “Safety and Efficacy of Remimazolam Compared With Placebo and Midazolam for Moderate Sedation During Bronchoscopy,” Chest 155, no. 1 (January 2019): 137–146. 30292760 10.1016/j.chest.2018.09.015

[18] H. Fang , Y. Zhang , J. Wang , et al., “Remimazolam Reduces Sepsis‐Associated Acute Liver Injury by Activation of Peripheral Benzodiazepine Receptors and p38 Inhibition of Macrophages,” International Immunopharmacology 101 (December 2021): 108331. 34810122 10.1016/j.intimp.2021.108331

[19] F. F. Buchanan , P. S. Myles , and F. Cicuttini , “Effect of Patient Sex on General Anaesthesia and Recovery,” British Journal of Anaesthesia 106, no. 6 (June 2011): 832–839. 21558068 10.1093/bja/aer094

[20] F. De Wit , A. L. Van Vliet , R. B. De Wilde , et al., “The Effect of Propofol on Haemodynamics: Cardiac Output, Venous Return, Mean Systemic Filling Pressure, and Vascular Resistances,” British Journal of Anaesthesia 116, no. 6 (June 2016): 784–789. 27199311 10.1093/bja/aew126

[21] J. Y. Choi , H. S. Lee , J. Y. Kim , et al., “Comparison of Remimazolam‐Based and Propofol‐Based Total Intravenous Anesthesia on Postoperative Quality of Recovery: A Randomized Non‐Inferiority Trial,” Journal of Clinical Anesthesia 82 (November 2022): 110955. 36029704 10.1016/j.jclinane.2022.110955

[22] K. Bakhshi , “An Open Randomized Study of the Effects of Intravenous Fluid Replacement During Day Case Anaesthesia,” Ambulatory Surgery 2, no. 1 (1994): 49–51.

[23] J. Gao , C. Yang , Q. Ji , and J. Li , “Effect of Remimazolam Versus Propofol for the Induction of General Anesthesia on Cerebral Blood Flow and Oxygen Saturation In Elderly Patients Undergoing Carotid Endarterectomy,” BMC Anesthesiology 23, no. 1 (May 2023): 153. 37142971 10.1186/s12871-023-02095-z PMC10157955

[24] C. K. Hofer , A. Zollinger , S. Bu¨chi , et al., “Patient Well‐Being After General Anaesthesia: A Prospective, Randomized, Controlled Multi‐Centre Trial Comparing Intravenous and Inhalation Anaesthesia,” British Journal of Anaesthesia 91, no. 5 (November 2003): 631–637. 14570783 10.1093/bja/aeg243

[25] J. Cremer , C. Stoppe , A. V. Fahlenkamp , et al., “Early Cognitive Function, Recovery, and Well‐Being After Sevoflurane and Xenon Anesthesia In the Elderly: A Double‐Blinded Randomized Controlled Trial,” Medical Gas Research 1 (2011): 9. 22146537 10.1186/2045-9912-1-9 PMC3231879

[26] E. T. Moro , F. C. O. Leme , B. R. Noronha , G. F. P. Saraiva , N. V. de Matos Leite , and L. H. C. Navarro , “Quality of Recovery From Anesthesia of Patients Undergoing Balanced or Total Intravenous General Anesthesia. Prospective Randomized Clinical Trial,” Journal of Clinical Anesthesia 35 (December 2016): 369–375. 27871559 10.1016/j.jclinane.2016.08.022

[27] D. Rörtgen , J. Kloos , M. Fries , et al., “Comparison of Early Cognitive Function and Recovery After Desflurane or Sevoflurane Anaesthesia In the Elderly: A Double‐Blinded Randomized Controlled Trial,” British Journal of Anaesthesia 104, no. 2 (February 2010): 167–174. 20042477 10.1093/bja/aep369

[28] S. Singh , A. Kumar , R. Mahajan , S. Katyal , and S. Mann , “Comparison of Recovery Profile for Propofol and Sevoflurane Anesthesia In Cases of Open Cholecystectomy,” Anesthesia: Essays and Researches 7, no. 3 (September 2013): 386–389. 25885989 10.4103/0259-1162.123259 PMC4173547

[29] B. Fredman , E. Zohar , A. Philipov , D. Olsfanger , M. Shalev , and R. Jedeikin , “The Induction, Maintenance, and Recovery Characteristics of Spinal Versus General Anesthesia In Elderly Patients,” Journal of Clinical Anesthesia 10, no. 8 (December 1998): 623–630. 9873961 10.1016/s0952-8180(98)00099-3

[30] M. Martikainen , “One‐Week Recovery Profiles After Spinal, Propofol, Isoflurane and Desflurane Anaesthesia In Ambulatory Knee Arthroscopy,” Ambulatory Surgery 8, no. 3 (July 2000): 139–142. 10856843 10.1016/s0966-6532(00)00052-4

[31] S. W. Nam , S. H. Do , J. W. Hwang , I. Park , I. Hwang , and H. S. Na , “Effects of Opioid‐Sparing General Anesthesia on Postoperative Nausea and Vomiting In Laparoscopic Gynecological Surgery,” Korean Journal of Anesthesiology 77, no. 6 (August 2024): 605–613. 39183170 10.4097/kja.24336 PMC11637591

[32] W. Cheng , Y. Cheng , H. He , et al., “Efficacy and Safety of Remimazolam Tosilate In Anesthesia for Short Otolaryngology Surgery,” BMC Anesthesiology 24, no. 1 (November 2024): 407. 39528975 10.1186/s12871-024-02790-5 PMC11552106

[33] A. Li , N. Li , L. Zhu , et al., “The Efficacy and Safety of Ciprofol Versus Propofol In Patients Undergoing Painless Hysteroscopy: A Randomized, Double‐Blind, Controlled Trial,” BMC Anesthesiology 24, no. 1 (November 2024): 411. 39533194 10.1186/s12871-024-02787-0 PMC11555848

[34] K. Q. He , T. T. Huang , M. Y. Tan , C. Gao , and S. Wang , “Efficacy and Safety of Ciprofol Versus Propofol as Anesthetic for Patients Undergoing Painless Colonoscopy,” Pain and Therapy 13, no. 6 (December 2024): 1633–1644. 39400664 10.1007/s40122-024-00662-x PMC11543975

[35] A. Gupta , M. Kullander , K. Ekberg , and C. Lennmarken , “Assessment of Recovery Following Day‐Case Arthroscopy: A Comparison Between Propofol and Isoflurane‐Based Anaesthesia,” Anaesthesia 50, no. 11 (November 1995): 937–942. 8678247 10.1111/j.1365-2044.1995.tb05923.x

[36] R. M. Engelman , “Mechanisms to Reduce Hospital Stays,” Annals of Thoracic Surgery 61, no. 2 (February 1996): s26–s29. 8572829 10.1016/0003-4975(95)01081-5

[37] K. Ohkushi , K. Fukuda , Y. Koukita , Y. Kaneko , and T. Ichinohe , “Recovery Profile and Patient Satisfaction After Ambulatory Anesthesia for Dental Treatment—A Crossover Comparison Between Propofol and Sevoflurane,” Anesthesia Progress 63, no. 4 (2016): 175–180. 27973936 10.2344/15-00012.1 PMC5157142

[38] U. Nilsson , M. Jaensson , K. Dahlberg , and K. Hugelius , “Postoperative Recovery After General and Regional Anesthesia In Patients Undergoing Day Surgery: A Mixed Methods Study,” Journal of Perianesthesia Nursing 34, no. 3 (June 2019): 517–528. 30470465 10.1016/j.jopan.2018.08.003

[39] L. Yu , Y. Dong , S. Shi , X. Liu , M. Wang , and G. Jiang , “Analysis of Postoperative Nausea and Vomiting In Patients With Lung Cancer Undergoing Thoracoscopic Surgery under General Anesthesia and Its Influencing Factors: An Observational Study,” BMC Surgery 24, no. 1 (October 2024): 316. 39415116 10.1186/s12893-024-02614-w PMC11484199

[40] G. Liu , J. Zhang , L. Zhang , L. Yuan , X. Wang , and D. Tursunmamat , “Comparison of Spinal Anesthesia and Local Anesthesia In Percutaneous Interlaminar Endoscopic Lumbar Discectomy for L5/S1 Disc Herniation: A Retrospective Cohort Study,” BMC Musculoskeletal Disorders 25, no. 1 (October 2024): 774. 39358751 10.1186/s12891-024-07898-w PMC11447979

[41] H. T. Duran , M. Kızılkaya , A. Aydinli , et al., “The Effect of Two Different Modes of Anesthesia Maintenance on Postoperative Delirium In Elderly Patients With Low Preoperative Mini‐Cog Score,” BMC Anesthesiology 24, no. 1 (October 2024): 350. 39354373 10.1186/s12871-024-02735-y PMC11443701

[42] Y. E. Joe , C. M. Kang , H. M. Lee , K. J. Kim , H. K. Hwang , and J. R. Lee , “Quality of Recovery of Patients Who Underwent Curative Pancreatectomy: Comparison of Total Intravenous Anesthesia Versus Inhalation Anesthesia Using the QOR‐40 Questionnaire,” World Journal of Surgery 45, no. 8 (August 2021): 2581–2590. 33881579 10.1007/s00268-021-06117-0

